# Shared patterns of species turnover between seaweeds and seed plants break down at increasing distances from the sea

**DOI:** 10.1002/ece3.893

**Published:** 2013-12-05

**Authors:** Carlos F D Gurgel, Thomas Wernberg, Mads S Thomsen, Bayden D Russell, Paul Adam, Jonathan M Waters, Sean D Connell

**Affiliations:** 1 The Environment Institute, Australian Centre for Evolutionary Biology and Biodiversity, School of Earth and Environmental Sciences, University of AdelaideDX 650-418, Adelaide 5005, South Australia, Australia; 2 Plant Biodiversity Centre, State Herbarium of South AustraliaGPO Box 1047, Adelaide 5001, South Australia, Australia; 3 Aquatic Sciences, South Australian Research and Development InstitutePO Box 120, Henley Beach 5022, South Australia, Australia; 4 UWA Oceans Institute & School of Plant Biology, University of Western AustraliaCrawley, 6009, Western Australia, Australia; 5 Department of Marine Ecology, National Environmental Research Institute4000, Roskilde, Denmark; 6 The Environment Institute, Southern Seas Ecology Laboratories, University of AdelaideDP 418, Adelaide 5005, South Australia, Australia; 7 School of Biological, Earth, and Environmental SciencesUniversity of New South Wales, Sydney, New South Wales, Australia; 8 Department of Zoology, University of OtagoDunedin, New Zealand

**Keywords:** Australia, biogeography, connectivity, herbarium, macroalgae, seed plants

## Abstract

We tested for correlations in the degree of spatial similarity between algal and terrestrial plants communities along 5500 km of temperate Australian coastline and whether the strength of correlation weakens with increasing distance from the coast. We identified strong correlations between macroalgal and terrestrial plant communities within the first 100 km from shore, where the strength of these marine–terrestrial correlations indeed weakens with increasing distance inland. As such, our results suggest that marine-driven community homogenization processes decompose with increasing distance from the shore toward inland. We speculate that the proximity to the marine environment produces lower levels of community turnover on land, and this effect decreases progressively farther inland. Our analysis suggests underlying ecological and evolutionary processes that give rise to continental-scale biogeographic influence from sea to land.

## Introduction

The search for concordant patterns of species distribution across multiple taxonomic levels, ecological systems and spatiotemporal scales is a fundamental goal of biogeographic research (Lomolino et al. 2010). For example, congruence among patterns of species distribution has historically been used to support the existence of overarching abiotic forces shaping evolution and biogeography throughout various levels of biological and ecological scales (e.g., vicariance, long distance dispersal; Nelson 1969; Croizat et al. 1974; Nelson and Platnick 1981).

Coastal (terrestrial) and marine ecosystems present ideal systems for elucidating ecological and evolutionary processes driving species distribution, community structure, and biodiversity. Yet, several noticeable differences exist between these two ecosystems as a consequence of the essential differences in their physical environment, markedly the relative prevalence of air or water, respectively. The extent to which species distribution is limited to a particular area is a result of a range of biotic and abiotic processes that vary in space and time, and these are also expected to differ markedly between terrestrial and marine ecosystems. In the marine environment, one of the main processes influencing species distribution refers to the presence, strength, physical attributes (e.g., temperature), and direction of boundary currents. For example, an increasing number of studies show how spatial patterns of marine biological similarity are often related to linear boundary currents, which characterize many coastal regions of the world (Waters et al. 2010; Coleman et al. 2011; Watson et al. 2011; Colgan and da Costa 2013). In marine systems, levels of current-mediated oceanographic connectivity can be an excellent predictor of regional biogeographic similarity at multiples levels of biological organization (e.g., Gaylord and Gaines 2000; Watson et al. 2011). The geographic range of terrestrial taxa, on the other hand, may be more strongly affected by a diverse array of nonlinear biotic and abiotic factors (e.g., habitat availability, climate, geology, geomorphology, and pedology; Clark et al. 1999; Cain et al. 2000; Moritz et al. 2000; Kinlan and Gaines 2003). In short, the larger magnitude and rate of dispersal not only of seeds and propagules but also of nutrients and materials in the marine environment promoted by the physical properties of constant and often directional water movement are expected to increase the scales of physical and biological connectivity among marine and nearshore communities compared with terrestrial and more inland ecosystems (Caley et al. 1996; Carr et al. 2003). Consequently, marine systems are often considered more “open”, connected and homogeneous compared with terrestrial systems. As a result, patterns of biological connectivity and species spatial turnover on land are expected to be more structured and heterogeneous than in the sea (Caley et al. 1996; Carr et al. 2003).

Temperate Australia presents an excellent system for marine and coastal biogeographic research. Australia's southern marine environments, for example, are characterized by strong patterns of biogeographic differentiation, as recognized by shifts in community composition (Waters et al. 2010), species distributional (e.g., Bennett and Pope 1953; Womersley and Edmonds 1958; Knox 1963; O'Hara and Poore 2000), and also by more recent phyto- and phylogeographic studies (Crisp et al. 2001; Waters 2008; Ayre et al. 2009; Li et al. 2013). These patterns are particularly influenced in the west, southwest and southern coasts by the Leeuwin Current (LC), the world's longest boundary current (Ridgway and Condie 2004; Waters et al. 2010). The LC also represents one of the most linear and geologically stable boundary currents in the planet (Phillips 2001).

In addition to the obvious direct effect of oceanography on the biogeography of marine benthic communities, the generally accepted role of marine-mediated dispersal (e.g., rafting) of terrestrial plants across long distances suggests that coastal oceanographic processes might also play a role in shaping the biogeography of seed plant communities closest to the coast (Hanski 1999; Kinlan and Gaines 2003; Stuessy 2007; Bellemain and Ricklefs 2008). However, very few studies have explored or quantified potential biogeographic links between marine and adjacent terrestrial ecosystems.

Here, we conducted comparative biogeographic analyses of regional species turnover between marine benthic macroalgal and terrestrial seed plant communities to test for the presence of shared patterns in spatial structure of community organization.

## Methods

### Data source

We built six taxonomically updated datasets based on distributional data for temperate Australian marine macroalgae (Rhodophyta, Chlorophyta, and Phaeophyceae, one dataset) and coastal seed plant species (Gymnosperms + Angiosperms sensu Crisp et al. 2001; five datasets) derived from the Australian Virtual Herbarium (AVH) database (summarized in Table 1); therefore, data were based on actual collection records. AVH provides access to information from over six million plant, alga, and fungi specimens held in the nine major state and territory herbaria in Australia. Data stored with these specimens provide a permanent historical record of the occurrence of a species at a particular place and time, are the primary resource for research on the classification and distribution of the Australian flora, and correspond to the most complete picture of the distribution of Australia's flora to date (The Council of Heads of Australasian Herbaria, 2013).

**Table 1:** Description of the six plant marine and terrestrial datasets used in this study. Specimen records were derived from Australian Virtual herbarium database. Each dataset is composed of 10 geographic IMCRA linear bioregions following the Australian western and southern coastline (as per Fig. 1). Flora is comprised of macroalgae for the marine environment and seed plants for the terrestrial environment

Environment	Marine	Terrestrial	Terrestrial	Terrestrial	Terrestrial	Terrestrial
Distance from the ocean	0 (∼50 km)	∼5–500 m (500 m)	500 m–2 km (1.5 km)	2–10 km (8 km)	10–50 km (40 km)	50–100 km (50 km)
Number of species	1118	4428	4189	5636	7819	6943
Number of records	40,136	70,095	46,499	99,068	257,209	154,663

**Figure 1:**
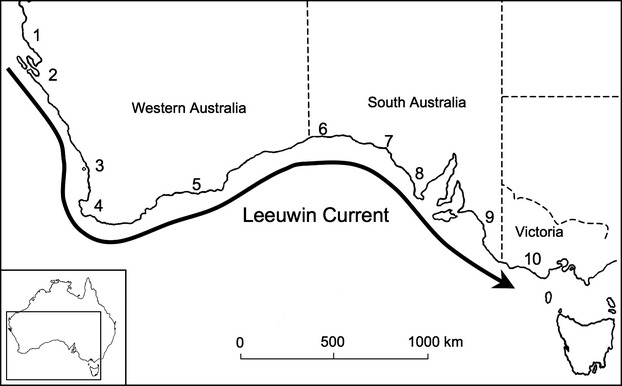
Map of southern Australia showing the 10 temperate IMCRA bioregions used in this study: 1 = Zuytdorp, 2 = Shark Bay, 3 = Central West Coast, 4 = Leeuwin-Naturaliste, 5 = WA South Coast, 6 = Eucla, 7 = Murat, 8 = Eyre, 9 = Coorong, and 10 = Otway. Insert: location of the study are in the Australian continent.

Each dataset was composed of species presence or absence across 10 distinct temperate IMCRA bioregions running parallel to the coastline (= Interim Marine Coastal Regionalization of Australia, Commonwealth of Australia 2005). Our analysis encompassed the western and southern Australian coast, from the Zuytdorp (WA) to the Otaway bioregion (southern Victoria) (Fig. 1). The uniform coastal orography along the western and southern coast of Australia favored our analysis by minimizing the putative effect of topographic relief on changes in community structure as empirically demonstrated elsewhere (i.e., North America where landscape heterogeneity tended to increase linearly with topographic relief, Riera et al. 1998). Therefore, we avoided data from the Australian's east coast due to the presence of The Great Divide Range, or the Eastern Highlands, Australia's most substantial mountain range. South Australian Gulf bioregions were also not included in the analysis to avoid problems created by the unique oceanography (inverted estuaries) and complex coastline geography of the region (i.e., 100 km inland to the Yorke Peninsula leads again to marine conditions seeing that such peninsula is less than 50 km in diameter).

We used the marine bioregionalization of Australia (rather than terrestrial) to compare marine and terrestrial plant assemblages followed the central aim of this study in testing for the presence of a marine signature driving homogenization of terrestrial plant communities closest to the ocean. As a tool for organizing spatial information, IMCRA regionalization helps to understand complex ecosystems, their specific management needs and plays a pivotal role in federal, state and local conservation programs (Spalding et al. 2006). Furthermore, IMCRA bioregions represent units of community characterization independent of spatial extent of coast or any unique terrestrial geomorphological or climatic feature. It is therefore relevant to test marine-related biogeographic hypotheses using IMCRA bioregions.

The superimposition of IMCRA bioregions over terrestrial data was achieved by drawing a line between each IMCRA boundary along the coast to the geographic central point of Australia. Bioregions were numbered sequentially from west to east (Fig. 1). Five distinct linear and adjacent “bands” (or concentric rings) of terrestrial vegetation assemblages at prescribed distances from the coast were then created, 0.00–0.5 km, 0.5–2 km, 2–10 km, 10–50 km, and 50–100 km (Table 1). These ad hoc distances correspond to those that also optimized the following co-occurring conditions: (1) a 500 meters coastal vegetation band containing enough area to secure a high number of specimen records influenced the most by oceanographic processes and (2) account for the fact that plant records and, to a certain extent, species diversity in Australia tend to be highest near the coast (where the climate is more mesic) and decrease toward inland (Hopper 1979). By increasing the area of the bands toward inland, we accounted for smaller number of historical records per area. Specimen GPS coordinates for all revised plant records were plotted over the 60 distinct communities (= 6 datasets × 10 bioregions each) using ArcGIS® software by ESRI (http://www.esri.com). In all datasets, taxonomic ranks below species level were collapsed into their respective species. Hybrids, incomplete and dubious identifications, and species represented by less than 3 records in AVH, were excluded from the analyses. The higher levels of biodiversity in our datasets reflect the inclusion of non-native species as well hence our analyses encompass the totality of plant biodiversity within the studied area.

### Data analysis

All multivariate data analyses were carried out in PRIMER 6.1.10 (Plymouth Routines in Multivariate Ecological Research, U.K.), and the routines mentioned refer to this program. Bray–Curtis dissimilarities were calculated from presence–absence data (= Sørensen similarity index) of macroalgal and land seed plant species, respectively, between each of the 60 plant communities. This index reflects pairwise composition dissimilarities between communities and bioregions (or similarity, depending on how you look at it). Ordination by principal coordinate analysis (PCO) was used to visualize patterns of similarity among regions for each plant dataset (= 6 ordinations). The presence of non-random spatial serial correlation in assemblage composition between successive and linearly organized bioregions was tested using RELATE. Consecutive bioregions were linearly organized eastward following the coastline, from 1 to 10 along the LC direction, as described in Figure 1. Seriation is a tool to test for spatial species turnover along a spatial or environmental gradient, or linear timelines (Brower and Kyle 1988; Clarke et al. 1993). Seriation is also a term used to define the serial change in community structure as a result of natural processes where mixed regions exist (Clarke and Warwick 2001). The index of seriation is given by Rho (*ρ*) and varies from −1 to +1 depending on the kind of correlation identified. The seriation index also assesses the extent to which samples follow a simple trend, or pre-established model (in this case linear long the coast from bioregion 1 to 10 as depicted in Fig. 1), with adjacent bioregions being the closest in composition, bioregion two distances apart being the next closest and so on, with bioregions from the first and last distance (1 and 10) differing the most. This index provides a *P*-value with the conventional 5% significance level.

RELATE was also used to assess the agreement between multivariate structure from the macroalgal dataset against each terrestrial plant dataset (i.e., at different distances from the coast). In this case, RELATE uses Spearman's rank to correlate two sets of multivariate data (i.e., distances matrices) where a coefficient of 1 is a perfect match between sample relationships. For example, a coefficient of 1 would occur if the ranks in the first dataset match the ranks of the second dataset (e.g., the two most western samples are the most similar, and the next two eastern samples are most similar, so on and so forth). As part of this process, product-moment correlations were used to test the relationship between mid-interval multivariate community distances and spatial serial correlation. As such, RELATE is analogous to a Mantel test (Mantel 1967).

Mean pairwise Søresen similarities between macroalgal (marine) and each seed plant communities (terrestrial) were calculated and ordered by geographically increasing distances from the coast to assess levels of multivariate structural similarity between the marine and terrestrial environments and produce a visualization on how these associations change as the distances between these two distinct environments progressively widens.

## Results

**Figure 2:**
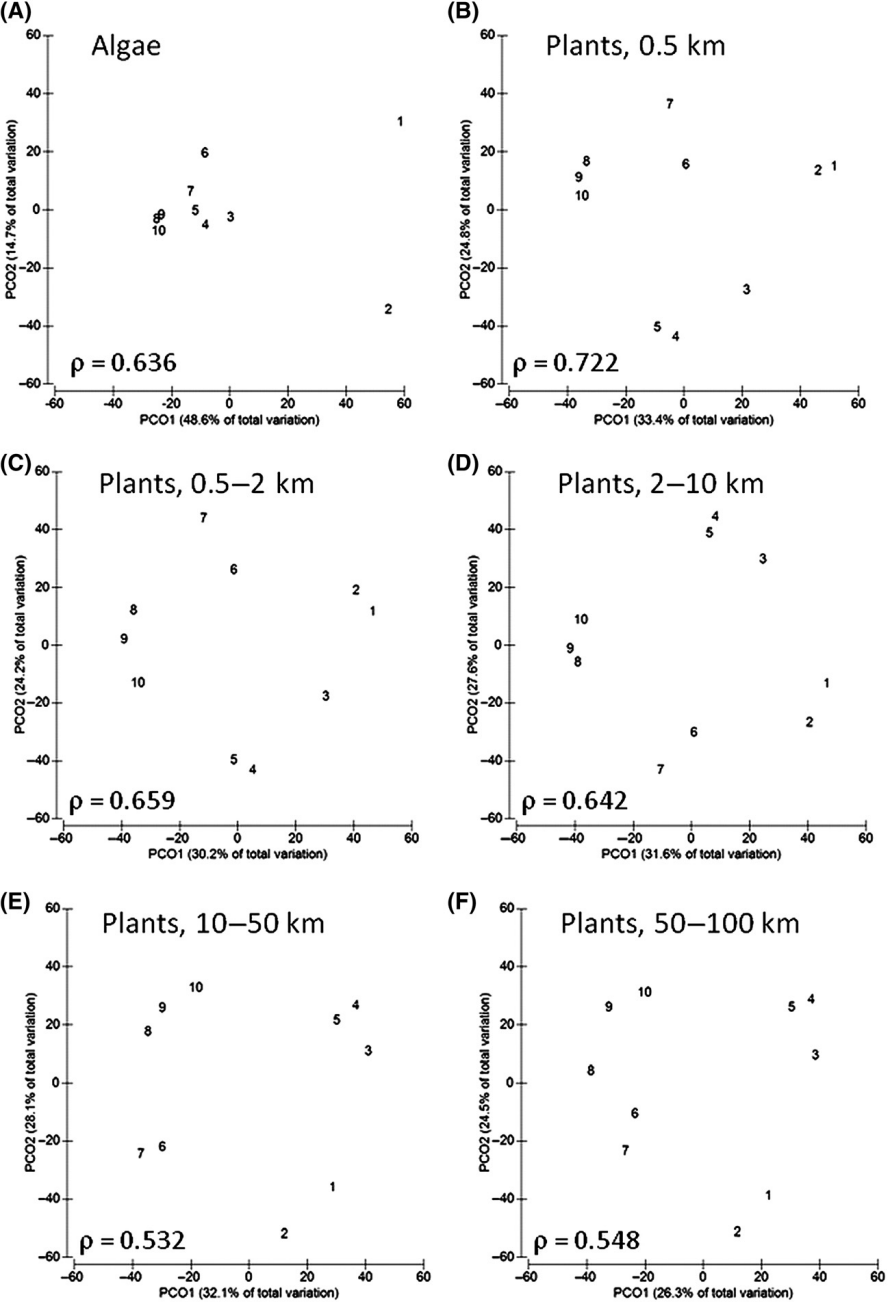
Six ordinations (A–F), each containing 10 marine or terrestrial plant assemblages from southern Australia (1–10). Each ordination corresponds to a distinct dataset, and plant assemblages were organized spatially following consecutive IMCRA bioregions with numbers found in each dataset corresponding to those in Fig. 1 (1 = Zuytdorp to 10 = Otway, west to east). Plant assemblages were also organized at different distance from the shore, that is, A: algae = zero distance, B: terrestrial plants 0–0.5 km from the water line inshore, C: 0.5–2.0 km. Rho (ρ) = Spearman's rank correlation between floristic composition and spatial distance (i.e., describes the strength of the presence of spatial seriation in the data). The presence of spatial seriation was statistically significant in all ordinations (ρ > 0.548, *P* < 0.03).

Ordination of six marine and terrestrial plant datasets and their respective 10 bioregions revealed broadly similar patterns regardless of their distances from the coast (Fig. 2). All ordinations are *in scale*. The macroalgal ordination (Fig. 2A), although similar to that produced for terrestrial plant datasets, is slightly more difficult to perceive as the multivariate dispersal of its 10 bioregions is smaller than those found for terrestrial plat communities (Fig. 2). This is also representative of a higher level of similarity among macroalgal communities across the ∼5500 km coastline compared with all terrestrial plant communities considered in this study. There was a significant spatial serial correlation of bioregions within all six datasets (*ρ* > 0.548, *P* < 0.03), and more importantly, the strength of this seriation weakened with increasing distance from the coast (*r* = −0.93, *P* = 0.008, *n* = 6).

**Figure 3:**
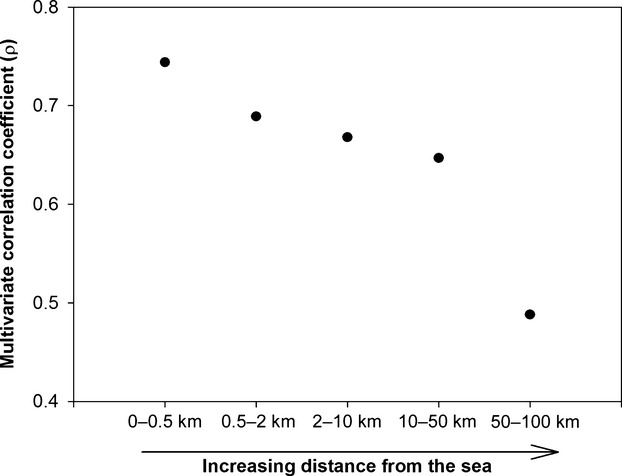
Correlation between multivariate patterns for marine (macroalgae) versus each of five terrestrial seed plant assemblages from western and southern temperate Australia, ordered by concentric rings of increasing distance from the coast (*x*-axis). All multivariate correlations are statistically significant.

Multivariate correlation coefficients between macroalgal and each terrestrial plant ordinations (RELATE) revealed a gradual and clear decline in the strength of agreement between ordinations with increasing distance inland (Fig. 3). The strength of this agreement between marine and terrestrial biogeographic spatial structure was strongest at the coast and dropped substantially beyond 50 km inland (Fig. 3). These multivariate correlation tests were statistically significant at *P* < 0.05

**Figure 4:**
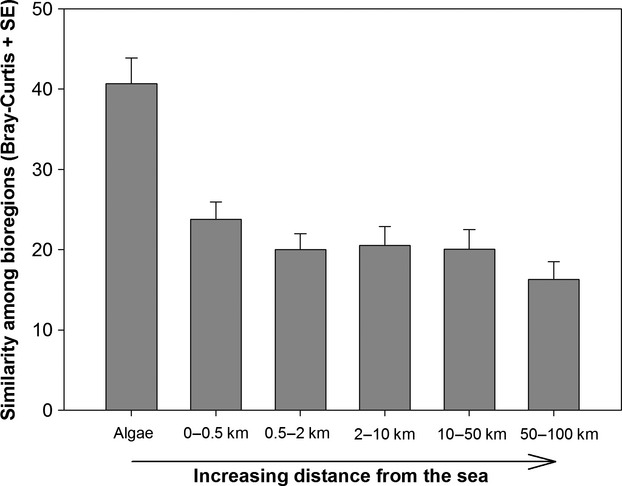
Mean pairwise Søresen similarities between marine and each terrestrial plant assemblage from southern Australia, ordered by geographic bands of increasing distance from the coast.

Patterns of species spatial turnover within and between datasets revealed that levels of assemblage similarity between bioregions were substantially higher (approximately twice as large) for marine algal assemblages than for terrestrial plant assemblages (Fig. 4). In addition, assemblages of land plants suggested a trend of decreasing levels of within dataset similarity with increasing distance inland, although this pattern was not statistically significant (*P* > 0.05) (Fig. 4).

## Discussion

The degree of similarity among algal communities along the Australian southwestern coastline was greater than the similarity among terrestrial plant communities. This was expected due to the higher degree of physical connectivity and environment homogeneity in the marine environment compared with those found in terrestrial ecosystems (Carr et al. 2003). However, our analyses also identified with different degrees of statistical significance, a clear overall pattern where the degree of along-coast spatial seriation and the spatial multivariate structural agreement between marine and terrestrial plant communities were all stronger closest to the coast and weakened with increasing distance inland.

We speculate that the maritime environment helps drive plant community homogenization and this driver decreases in strength with increasing distance from the coast. Several processes might be acting independently or in tandem, many of which influenced by maritime processes, to promote greater dispersal and lower levels of beta diversity among marine and coastal plant species and communities, respectively. Evidence on how direction, flow type, and strength of boundary currents act as dispersal agents comes from both classical and more recent marine biogeographic studies (Maxwell and Cresswell 1981; Banks et al. 2007; Waters et al. 2010). For example, it has been shown that the LC is capable of dispersing tropical marine fauna all the way into the temperate realms of southern Australia and the Great Australian Bight (Maxwell and Cresswell 1981). Higher connectivity among terrestrial plant communities along the western Australian coastline, however, may therefore be facilitated by increased seed dispersal via directional coastal currents, which we call part of a marine signature on land. The likelihood that seeds originated farther inland make their way to the coast to be dispersed along shore then back inland is far smaller than for seeds generated close to coastal areas. Evidently, this conjecture needs to be tested against a range of other explanatory, and not necessarily mutually exclusive, competitive hypotheses such as the influence of changes in soil type, elevation, changes in rainfall patterns, dispersal syndromes, and climate along the same coastal-land gradient. Soil types in particular have been considered a major determinant of plant community structure in southwestern Australia (Hopper 1979). Nevertheless, to pin point the exact drivers of the geographic patterns herein revealed was not the main objective of this study.

In higher plants, dispersal of individuals happens mostly as seeds (Levin et al. 2003). Even though seed long distance dispersal has been mostly attributed to bird-mediated transport, when oceanic currents have a direct (i.e., act as carriers of the actual seeds through space) or an indirect role (i.e., in ameliorating abiotic conditions across space so dispersed seeds can establish themselves over larger areas), they seem to become a major player in explaining plant dispersal (e.g., Trieste and Sierens 2013 and references therein).

Understanding continental-scale associations may provide insights for future work to account for the origin and maintenance of these patterns. Tests for the influence of other co-occurring non-marine processes in shaping seed plant species turnover (e.g., edaphic gradients, precipitation) may assist in our understanding of coastal plant biogeography, including community dynamics, genetics, and evolution. Not only historical (e.g., paleoclimatic change, geomorphology, and variations in sea level) but also contemporary marine factors such as maritime climate, oceanographic connectivity, direct or indirect marine-driven dispersal, might be playing a role in shaping terrestrial floristic composition and community spatial turnover. In reality, marine-influenced seed plant dispersal has been documented for a range of higher plant species (e.g., Barber 1970; Heyligers 1983, 1984). In addition, many if not all strand species in temperate Australia (plant species unique of nearshore habitats) are introduced. In that case, a better understanding of how marine-driven connectivity influences plant dispersal and community structure becomes a relevant factor for conservation practices and the protection of natural living resources (e.g., management of invasive species).

It is possible that the higher levels of similarities found between macroalgal bioregions compared with those observed across all terrestrial plants, and the non-significant trend of decreasing levels of community homogenization with increasing distance inland might also be partly linked to the underlying direct role of oceanographic distributors of propagules, which in turn are tightly regulated by type and direction of major boundary current flow (i.e., Gaylord and Gaines 2000; Kinlan and Gaines 2003). The farther inland plant specimens are the less likely they would benefit from longer-range dispersal by this means. Also, the maritime environment can influence the indirect role of other potential seed dispersers such as seashore birds. Coastal and marine bird species tend to be more mobile than their terrestrial counterparts, producing bigger connectivity in nearshore plant communities (Kinlan and Gaines 2003). Interestingly, boundary currents have been shown to produce a biogeographic imprint on coastal bird populations and their genetic structure (e.g., the seaside sparrow *Ammodramus maritimus*, Avise and Nelson 1989). Furthermore, it has been suggested that Australia seabirds play a pivotal role in long distance dispersal of terrestrial plant along coastal environments (Heyligers 1995). All these non-independent processes help to explain the results found in this study and point out to the relevant direct and indirect roles marine processes have in shaping coastal plant biogeography.

In conclusion, biogeographers have recognized continental-scale effects of maritime climate on plant communities in several parts of the world. Examples of this phenomenon include the role of the cold Benguela Current in creating the desert climate in southwest Africa (van Zinderen Bakker 1975), the cold Humboldt Current in facilitating the Atacama Desert (Gutiérrez et al. 1998), and the warming effect of the Gulf Stream on the northern European coast (Ehrlich and Roughgarden 1987). We propose a marine–terrestrial link across the Leeuwin Current, which represents one of the most linear and geologically stable boundary currents of the ocean. The recognition of the presence of lower alongshore plant community species turnover can also have strong implications for the design and management of conservation units where higher levels of connectivity are sought after (Crooks and Sanjayan 2006).
